# Towards computer-assisted TTTS: Laser ablation detection for workflow segmentation from fetoscopic video

**DOI:** 10.1007/s11548-018-1813-8

**Published:** 2018-06-27

**Authors:** Francisco Vasconcelos, Patrick Brandão, Tom Vercauteren, Sebastien Ourselin, Jan Deprest, Donald Peebles, Danail Stoyanov

**Affiliations:** 1 Wellcome / EPSRC Centre for Interventional and Surgical Sciences Centre For Medical Image Computing, University College London, London, UK; 2 Department of Obstetrics and Gynecology, University Hospitals Leuven, Louvain, Belgium; 3 Department of Obstetrics and Gynecology, University College London, London, UK

**Keywords:** Twin-to-twin transfusion syndrome (TTTS), Endoscopy, Deep learning, Workflow segmentation

## Abstract

**Purpose:**

Intrauterine foetal surgery is the treatment option for several congenital malformations. For twin-to-twin transfusion syndrome (TTTS), interventions involve the use of laser fibre to ablate vessels in a shared placenta. The procedure presents a number of challenges for the surgeon, and computer-assisted technologies can potentially be a significant support. Vision-based sensing is the primary source of information from the intrauterine environment, and hence, vision approaches present an appealing approach for extracting higher level information from the surgical site.

**Methods:**

In this paper, we propose a framework to detect one of the key steps during TTTS interventions—ablation. We adopt a deep learning approach, specifically the ResNet101 architecture, for classification of different surgical actions performed during laser ablation therapy.

**Results:**

We perform a two-fold cross-validation using almost 50 k frames from five different TTTS ablation procedures. Our results show that deep learning methods are a promising approach for ablation detection.

**Conclusion:**

To our knowledge, this is the first attempt at automating photocoagulation detection using video and our technique can be an important component of a larger assistive framework for enhanced foetal therapies. The current implementation does not include semantic segmentation or localisation of the ablation site, and this would be a natural extension in future work.

## Introduction

Twin-to-twin transfusion syndrome (TTTS) is a disease affecting identical twin pregnancies. It is caused by abnormal vessels in the placenta that disproportionately transfuse blood from one twin to the other. The recipient of excessive blood is at risk of significant complications including heart failure, while the second foetus is affected by slower than normal growth. The overall outcome is dismal for both if the condition is left untreated. TTTS can be treated by coagulating the abnormal vessels in the placenta, interrupting the excessive blood flow from one twin to the other. Foetal surgery for TTTS involves the fetoscopic identification of anastomosing vessels on the unique twin placenta and laser photocoagulation [14]. However, the current procedure has several significant challenges: inability for correct orientation of endoscope and laser in case of anterior placenta [4, 9]; incomplete visualisation in case of turbid amniotic fluid or small vessels [11]; uncertainties with classification of arteries and veins based on oxygen content to guide sequential lasering [12]; instability of the image without motion compensation when carried out at close proximity to prevent inadvertent ablation or tissue contact and fetoplacental haemorrhage. Computer-assisted interventions (CAI) can offer potential solutions to some of these challenges. To achieve this, it is essential to use algorithms for extracting information from the the fetoscope, which is the only sensor within the in utero environment.

**Fig. 1:**
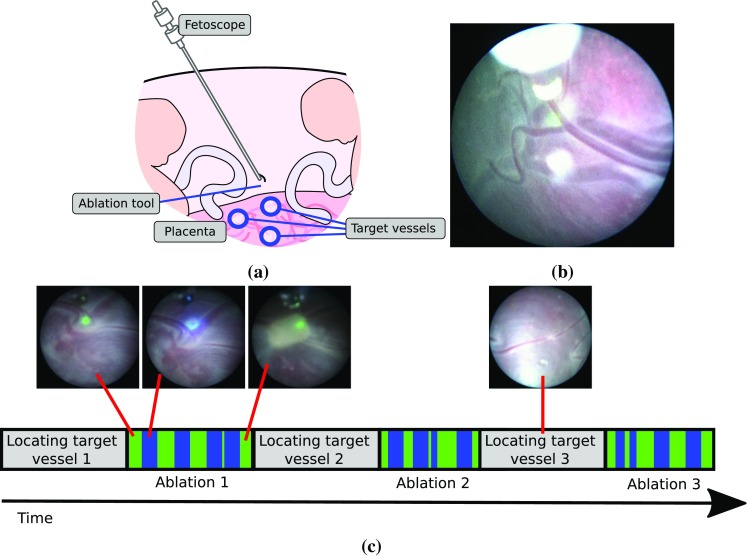
TTTS laser ablation therapy. **a** This procedure aims at coagulating a series of abnormal vessels in the placenta using a laser ablation tool inserted on a fetoscopic camera **b** fetoscopic image with the laser ablation tool visible and the placenta in the background; **c** timeline of a TTTS laser ablation procedure

**Fig. 2:**
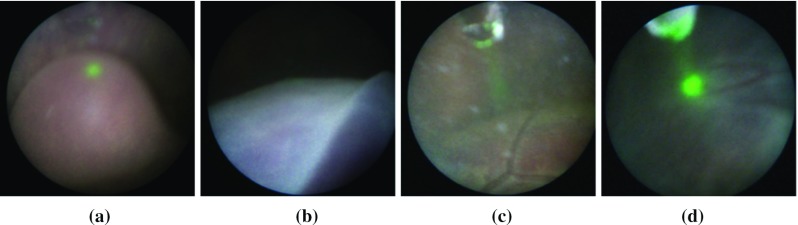
Examples of variability in fetoscopic images. **a** Out of focus scene; **b** occlusion by the umbilical chord; **c** amniotic fluid with high turbidity; **d** dim lighting conditions

TTTS interventions are performed using a fetoscopic camera with a retractable fibre-based laser ablation tool driven through a small working channel in the fetoscope. The surgeon navigates the fetoscope to sites on the placenta, identifying vascular structures and successively photocoagulating each of the abnormal vessels and targets them with the laser (Fig. 1c). The precise site for applying energy with the laser is controlled by the pose of the fetoscope as well as the insertion depth of the laser fibre. When inserted sufficiently, the ablation tool tip can be observed at the edge of the fetoscopic image as shown in Fig 1b. To assist the surgeon and indicate the point of energy delivery, the laser emits a low power projection that enables the surgeon to accurately aim at target vessels. Despite the fact that focused light from the laser ablation fibre provides very salient image features, especially in proximity with the placenta surface, there is a very high variability in image appearance between different patients, fetoscope hardware models, and different placental regions in intra- and inter-patients. This makes automatic video processing a challenge [2, 5, 6, 13] because complex appearance can occur due to a number of cases, for example: the fetoscope is not focused due to very dynamic changes in depth within the scene (Fig. 2a); the surgery is performed within a highly dynamic environment with foetuses that can move unpredictably and often occlude the camera field of view (Fig. 2b); the surgical environment is immersed in amniotic fluid, which becomes more turbid as the gestation evolves and degrades visibility (Fig. 2c); additionally, there is a very high variability in fetoscope light sources that can range from intense light with specularities on the placenta surface, to very dim lighting conditions (Fig. 2d). These factors render vision driven analysis of fetoscopic video a significant challenge and make it difficult to automatically detect events of interest, such as when the laser is actively photocoagulating.

Deep learning architectures for classification and semantic segmentation have been shown to be extremely effective in recent years. In minimally invasive surgery (MIS), such approaches have demonstrated to be the most effective for detection and classification of surgical instruments [7, 10] and even for detecting abnormal tissue structures [1] or segmenting surgical video into semantic steps [15]. In this paper, we take a similar approach and leverage the power of convolutional neural networks (CNNs) and their generalisation capabilities to handle high image variability in order to detect ablation during TTTS. This is the first time automation of recognition has been attempted in fetoscopic video.

Our contributions can be summarised as follows:

- We design CNN-based methods for detecting the occurrence of ablation in fetoscopy videos while taking into account the specificities of TTTS ablation therapy and its impact on the characteristics of image appearance.
- We demonstrate the generalisation ability of our method by testing it on large sequences with appearance characteristics that are significantly different to the training data.
- We use our trained CNN to indirectly infer the occurrence of other events during the surgical procedure that would not allow ablation to occur in the immediate future, including occlusions, large distance to the placenta, and inactive state of laser tool. This information is obtained without the necessity of annotating these events in the training data and can be used to increase the accuracy of ablation detection.

## Problem formulation

In this paper, our aim is to identify ablation during fetoscopic video and use this result to automatically segment different actions performed during a TTTS laser surgery using fetoscopic videos of the whole procedure. More specifically, we propose to separate surgical activity into three different classes. *Targeting*: The fetoscope is in close proximity to the placenta and could possibly start performing ablation in the immediate future. The laser ablation tool projects a green light circle on the surface of the placenta which indicates where its currently aiming at. *Ablation*: A vessel is being coagulated. The projected laser light increases in intensity and changes to a blue colour. The targeted vessel progressively changes in appearance, turning into a white coagulated blob. *Other*: Scenes where laser ablation could not possibly occur in the immediate future. The ablation tool is not targeting any surface, due to the fetoscope being too far from the placenta, occlusions, or the laser tool being turned off.

**Fig. 3:**
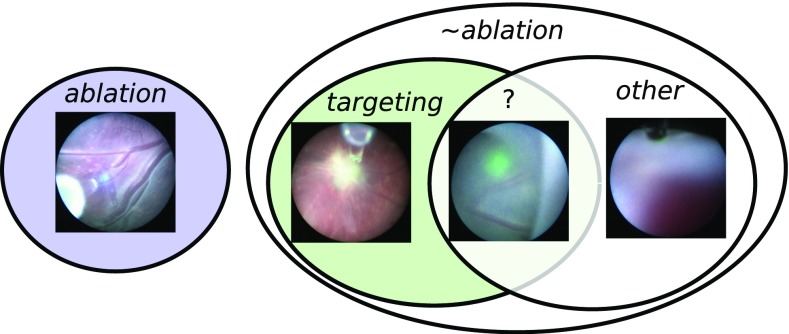
Proposed classification labels. The distinction between *targeting* and *other* is not clear-cut

The most easily distinguishable label is *ablation* due to the blue light emission and the changes in appearance of the targeted vessel. During *targeting*, the laser emits a green light that is projected on the placenta. However, the distinction between *targeting* and *other* is not clear-cut (Fig. 3), as the surgeon may move the fetoscope away from the placenta without turning off the green laser pointer. In such cases, it becomes difficult to establish objective criteria to separate the two labels. This poses the additional problem of how to objectively annotate groundtruth data that belong to the *targeting* and *other* classes while avoiding ambiguous training labels.

**Fig. 4:**
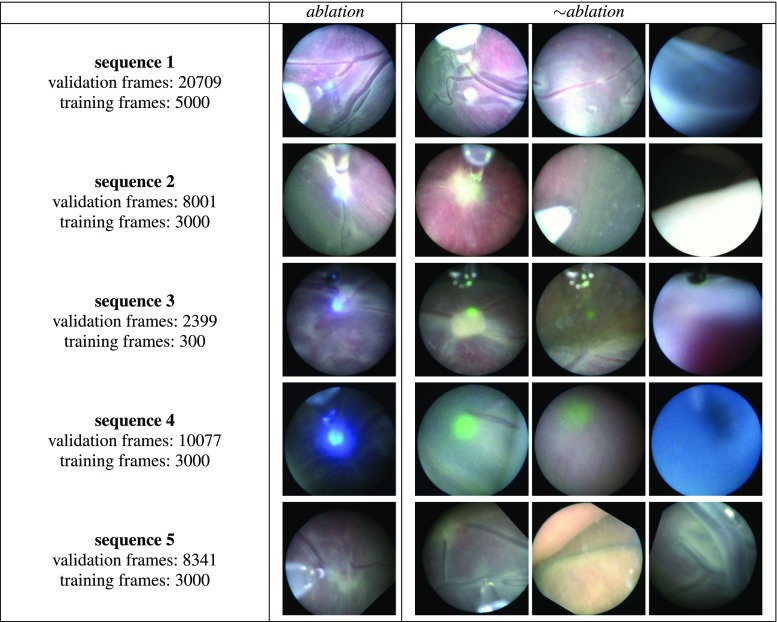
Training and validation data from five different TTTS laser ablation procedures

There is temporal information that might help performing this task: the considered actions should span over consecutive sequences of frames to guarantee temporal continuity; *ablation* sequences should be temporally adjacent to *targeting* sequences; changes in appearance of the placenta surface might indicate the occurrence of ablation; and finally, the motion patterns of the laser tool and projected light are significantly different from the scene motion. However, in this paper we only consider classification based on the appearance of each frame independently.

## Methods

Our study considers videos obtained from five different TTTS ablation procedures (Fig. 4). The sequences have significantly different appearances between them: sequences 1 and 2 have relatively bright lighting, sequence 3 has a higher turbidity than normal, sequence 4 has a weaker light source, and in sequence 5 the projected light from the laser pointer is not visible in most of the frames (ablation is still noticeable from appearance changes in the ablation tool itself).

In order to avoid the labelling ambiguity mentioned in the previous section, we manually annotate the data with only two labels: *ablation* and $\sim$*ablation*. These are easily distinguishable by visually inspecting the colour and intensity of the laser emitted light and are relatively fast to perform on thousands of frames. We annotated a total of 49527 frames. A complete TTTS ablation procedure contains a significantly larger amount of $\sim$*ablation* frames than *ablation* frames. This class imbalance, if not properly addressed, causes a classification bias towards $\sim$*ablation* labels. We therefore randomly subsample the complete sequences in order to create training datasets with an equal number of *ablation* and $\sim$*ablation* labels. Figure 4 details the number of training frames extracted from each sequence.

**Fig. 5:**
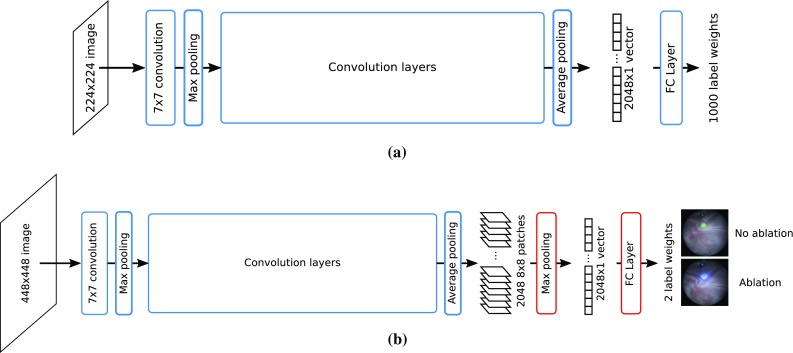
Convolutional Neural Network architecture. **a** Original ResNet with $224\times 224$ input images; **b** modified ResNet with an additional Max Pooling layer and $448\times 448$ input images

When the laser ablation tool is exposed its orientation on the fetoscopic images will broadly remain the same for a single ablation procedure. However, this fixed orientation changes arbitrarily between different procedures. Our training datasets include sequences from few procedures, and since we do not want our algorithm to over-fit the label classification to these particular orientations of the ablation tool, we perform a random rotation on all images of our training datasets.

### Binary classification network

We first consider the simpler problem of binary classification between *ablation* and $\sim$*ablation* labels.

We use the Residual Network (ResNet) architecture [8], which won the 2015 championship on three ImageNet competitions image classification, object localisation, and object detection. ImageNet [3] is a computer vision classification dataset containing non-medical images belonging to 1000 different labels. While previous CNN architectures usually suffer from over-fitting and slow convergence issues if the number of convolution layers is too high, ResNet has the ability to adapt well to the dimensionality and complexity of different problems while maintaining a high number of convolution layers. This makes it suitable to our problem, since we want to use a well performing CNN initialised on a large-scale dataset with very high variability, and adapt it to a problem with with only 2 labels and that deals with datasets that are limited in its appearance to laser ablation fetoscopy scenes.

We use the publicly available ResNet with 101 layers pre-trained on ImageNet as an initialisation (Fig. 5a). We then fine-tune the weights of all layers in this network using our training datasets. This network works with $224\times 224$ input images; however, our captured fetoscopic images are significantly larger. We test different approaches to deal with the image size difference: downsampling the input images to a $224\times 224$ resolution; or resizing them to a $448\times 448$ resolution (very close to their original size) and modifying the final ResNet pooling layer so that it properly connects the higher resolution images to the final fully connected (FC) classification layer. We test two different pooling modifications: increasing the kernel size of the ResNet averaging pooling layer; maintaining the kernel size of ResNet’s averaging pooling layer and adding a max pooling layer. Note that this final modification acts as a single pooling layer that performs a maximum over averages operation.

For both approaches, the fully connected (FC) final layer of the original ResNet is replaced to output two labels instead of 1000. The network is fine-tuned for a duration of 30000 iterations, with a learning rate of $1\times 10^{-5}$ and a batch size of four images.

### Complete classification pipeline

In order to further separate the $\sim$*ablation* labels into *targeting* and *other*, we must first establish a definition for these labels. We propose to define *targeting* frames as being images with the same appearance as the *ablation* frames, except all the salient blue features are green instead. In other words, a *targeting* image is such that if we swap its blue and green channels, it would be classified as *ablation*, if given as input to an algorithm that is able to reliably estimate *ablation* labels. Therefore, our complete classification algorithm consists in running the same binary classification CNN on both the original image and its version with swapped green and blue channels, ending up with two binary classifications for each frame: {*ablation*,$\sim$*ablation*} and {*targeting*,$\sim$*targeting*}. If an image is classified as both $\sim$*ablation* and $\sim$*targeting* we attribute it the label *other*. If an image is classified as both *ablation* and *targeting* we attribute it the label with the maximum ResNet score. Note that this last step has an impact on the results of the binary classifier by re-labelling some *ablation* frames to $\sim$*ablation*. In the experimental section, we show that the re-labelled frames are mostly false positive *ablation* detections, and therefore, our complete classification pipeline has a filtering action that provides a higher accuracy in ablation detection than a binary classifier.

## Results

We compare different ResNet-based algorithms for ablation detection and compare it against more classic SVM classification to demonstrate the non-trivial nature of this classification problem. The following algorithms are tested:

- *SVM+Hist* Support vector machine (SVM) classifier with a Gaussian kernel function. We discretise the HSV colour space into 4096 bins ($16\times 16 \times 16$) and perform PCA to reduce image features to the most discriminant 1000 dimensions. We also tried to remove the V component before building histograms, to test whether ablation appearance is independent from the overall scene illumination intensity. However, this lead to worse detection results.
- *ResNet+AVG* CNN binary classifier that increases the kernel size of the final average pooling layer of the ResNet architecture and uses $448 \times 448$ images as input.
- *ResNet+MAX* CNN binary classifier that adds an extra max pooling layer to the ResNet architecture and uses $448 \times 448$ images as input.
- *DS+ResNet* CNN binary classifier that downsamples input images to a $224 \times 224$ resolution and uses the original ResNet architecture.
- *Filtered ResNet* Our complete classification pipeline as described in Sect. 3.2. Note that this method can use any of the previous three algorithms as a basis for binary classification (Filtered ResNet+AVG, Filtered ResNet+MAX, Filtered DS+ResNet).

We use leave-one-out cross-validation to test the classification algorithms on each of the five datasets. The classification performance of the different methods is quantified in Table 1 by their precision (*p*), recall (*r*), and F-measure $f_{1}= 2/( 1/r + 1/p)$. In this paper, we use the $f_{1}$ an overall performance metric since it weights both precision and recall.

### Binary classification

From Table 1, we can observe that SVM+Hist is only able to perform well on sequence 4. Between the basic binary CNN classifiers, ResNet+AVG provides the best results. The best performance is generally obtained on sequence 4 which is characterised by its dark lighting conditions. This makes the ablation laser light the dominant feature in the image. On the other hand, the worst case is sequence 5 in which the ablation laser light projection is too weak to be visibly projected on the placenta surface.

**Table 1:** Precision (*p*), recall (*r*), and F-measure $f_{1}= 2/( 1/r + 1/p)$ in ablation detection for every classification method

Validation sequence		1	2	3	4	5
Hist+SVM	*p*	0.35	1.00	0.11	0.83	0.15
	*r*	0.41	0.02	0.32	0.90	0.12
	$f_{1}$	0.38	0.04	0.16	0.87	0.13
ResNet+AVG	*p*	0.91	0.80	1.00	0.98	0.65
	*r*	0.96	0.94	0.82	1.00	0.51
	$f_{1}$	**0.93**	0.86	**0.90**	**0.99**	0.57
ResNet+MAX	*p*	0.86	0.63	1.00	0.67	0.89
	*r*	0.98	0.98	0.78	1.00	0.47
	$f_{1}$	0.91	0.86	0.87	0.80	**0.61**
DS+ResNet	*p*	0.83	0.79	1.00	0.98	0.74
	*r*	0.97	0.96	0.77	0.96	0.51
	$f_{1}$	0.90	0.86	0.87	0.97	0.60
Filtered ResNet+AVG	*p*	0.91	0.85	1.00	0.98	0.65
	*r*	0.96	0.94	0.82	1.00	0.51
	$f_{1}$	**0.93**	0.90	**0.90**	**0.99**	0.57
Filtered ResNet+MAX	*p*	0.86	0.85	1.00	0.94	0.89
	*r*	0.98	0.98	0.78	1.00	0.47
	$f_{1}$	0.91	0.91	0.87	0.97	**0.61**
Filtered DS+ResNet	*p*	0.84	0.97	1.00	0.98	0.74
	*r*	0.97	0.95	0.77	0.96	0.51
	$f_{1}$	0.90	**0.96**	0.87	0.97	0.60

Bold indicates the highest $f_1$ scores for each validation sequence

### Complete classification

**Fig. 6:**
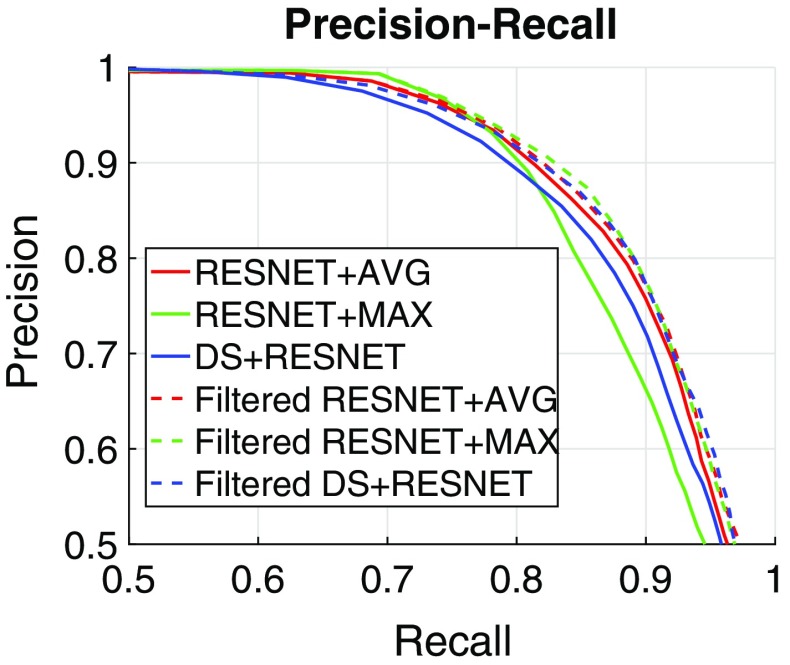
Cumulative results: precision-recall curve

**Fig. 7:**
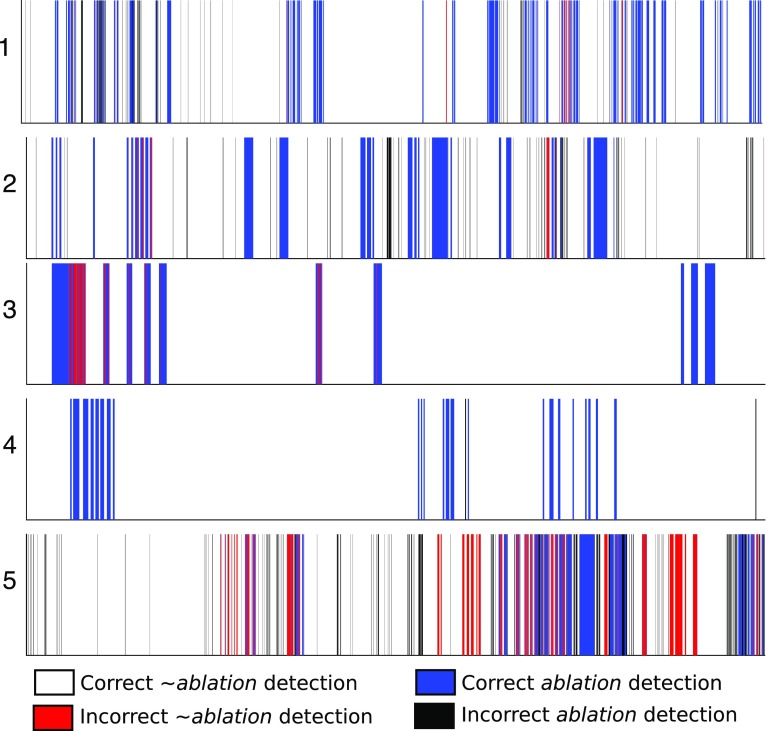
Ablation detection timelines of Filtered DS+ResNet for the five sequences

**Fig. 8:**
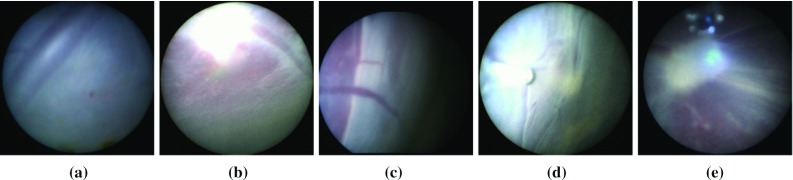
Examples of failed detections: **a** False positive on sequence 1; **b** false positive on sequence 2; **c** false positive on sequence 5; **d** false negative on sequence 2; false negative on sequence 3

The filtering approach based on our complete classification pipeline improves all three CNN methods on dataset 2 and ResNet+MAX on dataset 4. On the remaining cases, the performance is either the same or marginally improved. Results are improved by increasing the precision at the cost of a slight decrease in recall, which is to be expected due to its filtering action on *ablation* labels. We also display in Fig. 6 the precision-recall curve for the three CNN-based algorithms, using the final labelling ResNet score as a varying parameter. It can be seen that filtering improves the overall performance of all CNN-based methods. Table 2 presents the cumulative results of the filtering approaches over all five validation sequences. Figure 7 displays a timeline with the filtered ResNet+MAX predictions of *ablation* and $\sim$*ablation* labels for the whole duration of all five sequences, representing both correct and incorrect detections. In Fig. 8, we display some examples of incorrect detections.

**Table 2:** Cumulative results: confusion tables

	Prediction	
Filtered ResNet+AVG	*ablation*	$\sim$ *ablation*
*ablation*	6037	954
$\sim$ *ablation*	1061	41475
Filtered ResNet+MAX	*ablation*	$\sim$ *ablation*
*ablation*	6054	866
$\sim$ *ablation*	1044	41563
Filtered DS+ResNet	*ablation*	$\sim$ *ablation*
*ablation*	6040	925
$\sim$ *ablation*	1058	41504

**Fig. 9:**
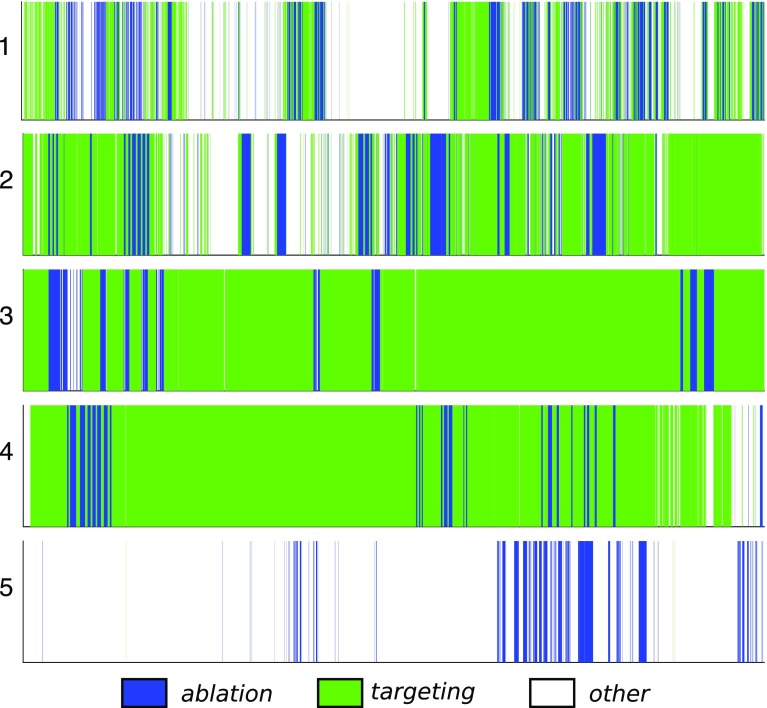
Complete classification timelines of Filtered DS+ResNet for the five sequences

We note that this increase in performance is obtained while simultaneously identifying a third label that enhances the qualitative descriptiveness of TTTS ablation timelines. Figure 9 displays results for the classification of the three labels: *ablation*, *targeting*, and *other*. We notice that in the two best performing sequences, 3 and 4, most of the $\sim$*ablation* frames are labelled as *targeting* with a few short sequences of *other* labels being spread throughout the sequences. On the other hand, the worst performing sequence (5) presents the largest sequences of *other* labels.

**Fig. 10:**
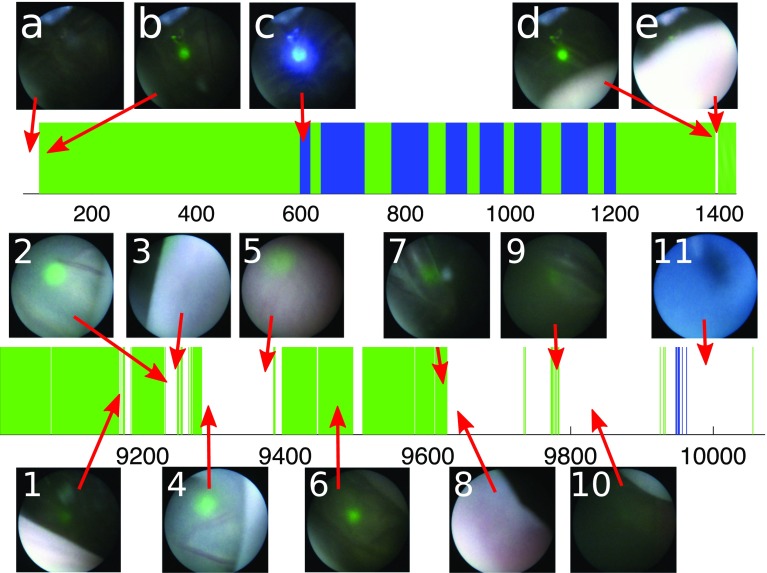
Zoomed in details from sequence 4 in Fig. 9: beginning of the ablation procedure above, and end of the ablation procedure below

Given that sequence 4 is the one with the most accurate ablation labels, we examine it in more detail to analyse the distinctions between the labels *targeting* and *other*, and understand to what type of events they correspond to in the TTTS ablation procedure. Figure 10 displays in more detail the beginning and the end of sequence 4. At the beginning of the procedure one can notice that before the laser is turned on (image a) the frames are classified as *other* and as soon as the laser is turned on the frames are classified as *targeting* (image b). It is also worthy of notice that a small sequence of *other* labels (image e) is due to the scene being occluded by the umbilical chord. At the end of the procedure, we can further analyse the behaviour of our classification network. There is a series of intermittent classifications between *targeting* and *targeting* which indicates an ambiguous sequence of frames. This is caused by the fetoscope being further away from the placenta (Image 1). The transition between Image 2 and Image 3 is caused by an occlusion with the umbilical chord. In image 4, the fetoscope is again starting to move away from the placenta causing the laser projection to be progressively less focused. When the fetoscope is furthest away from the placenta (Image 5), the laser pattern is significantly less visible. Image 6 is an easy classification example where the laser is clearly visible again. Image 7 displays a case where the fetoscope is very close to the placenta, but the laser light is not visible due to the angle at which the fetoscope is positioned. Image 8 belongs to the very ending of the procedure where the camera slowly moves away from the placenta until it is completely removed from the patient. The laser only focuses on the placenta for very short periods of time (Image 9), but for the most part the laser is not visible at all (Image 10). Image 11 depicts an interesting sequence where the camera is removed from the patient through an entirely blue cannula, causing a few false positive detections.

## Discussion

From our results we can observe that a simple SVM solution based on colour histograms is not enough to predict ablation labels. All CNN-based methods perform better with darker environments (sequence 4), and worst with weak ablation laser lighting (sequence 5). Additionally, our complete pipeline seems to detect more *targeting* labels in datasets where the ablation laser is more visible. On one hand, this makes sense since we expect that the tool is mostly ready for ablation once its laser light is clearly projected on the placenta, showing sufficient proximity. On the other hand, this might not account for cases where the surgeon has to deal with poor visibility conditions throughout the whole procedure. This is an important information to take into account when selecting data for future expansions of the training dataset.

The fact that the Filtered ResNet approaches provide the best results shows that our heuristic approach to distinguish between *targeting* and *other* labels produces meaningful results. Our established prior that images similar to ablation with a green pattern belong to a label on its own provides useful information that increases the accuracy of ablation detection without the need of additional image annotation. This is further confirmed by the qualitative analysis on Fig. 10 where we show that, when ablation detection is accurate, *other* labels correspond to meaningful events (occlusions, laser turned off, camera too distant from placenta, etc). Obviously, we expect that in datasets where ablation detection is less successful (sequence 5), the frames classified as *other* will be also less meaningful and descriptive.

## Conclusions

In this paper, we have shown that automatic detection of surgical action in fetoscopic video is feasible. Our focus was on ablation detection from video-based analysis as a precursor capability for more advanced CAI capabilities. While hardware solutions may be possible, a pure vision approach is appealing within the context of broader visual scene understanding. This problem is seemingly easy to solve due to the emission of a coloured light from the laser fibre but is not the case in practice due to the complexity of the environment during TTTS. Therefore, simple approaches such as classification on colour histograms failed to provide any meaningful results.

Our results indicate that the deep learning approaches perform beyond the capabilities of hand crafted techniques for an environment that presents many dynamic challenges. We also show that it is possible to incorporate heuristic prior information to increase detection accuracy and potentially provide useful information to distinguish between finer segmentation labels. Additionally, the recognition of different vessel regions, the detection of coagulated vessels, as well as motion analysis could potentially be useful to distinguish between actual targeting periods immediately before an ablation and other different tasks that are very similar in image appearance, such as vessel inspection after ablation, and navigation between two target vessels. We also need to experiment further with different types of scopes and ablation tools in order to further expand the training set.
